# Improving collegiate student-athletes’ well-being: exploring the roles of openness to experience, knowledge sharing and perceived coaching effectiveness

**DOI:** 10.3389/fpsyg.2023.1191622

**Published:** 2023-07-27

**Authors:** Peihao Ni, Ligang Feng

**Affiliations:** ^1^Department of Physical Education, Woosuk University, Jeonju, Republic of Korea; ^2^Schol of General Education, Shenyang City University, Shenyang, China

**Keywords:** collegiate student-athletes, personality traits, stressors, sustainable career development, knowledge sharing, coaching effectiveness

## Abstract

**Introduction:**

Collegiate student-athletes often encounter various stressors stemming from academic study and athletic training, which can potentially have negative effects on their well-being. This study investigates how collegiate student-athletes’ openness to experience and their engagement in knowledge sharing influence their well-being, as well as the moderating role of perceived coaching effectiveness.

**Methods:**

To examine these relationships, we propose and test a conceptual framework using an online survey conducted among collegiate student-athletes from a southeastern province of China. The participants consisted of 484 collegiate student-athletes who voluntarily participated in the study. We used regression analysis and mediation analysis to test the proposed relationships among the variables.

**Results:**

Openness to experience has a positive impact on knowledge sharing (β = 0.552, *p* < 0.05); knowledge sharing with peers positively affects collegiate student-athlete well-being (β = 0.415, *p* < 0.05) and mediates the relationship between openness to experience and collegiate student-athlete well-being (β = 0.086, *p* < 0.05). Perceived coaching effectiveness positively moderates the relationship between openness to experience and knowledge sharing (β = 0.170, *p* < 0.05).

**Discussion:**

Our study contributes to the collegiate student-athlete literature by shedding light on the factors that influence their well-being, with insights that bear important managerial implications for universities and coaches.

## Introduction

A collegiate student-athlete refers to a full-time or part-time student who is registerred in a university to participates in organized and competitive athletic programs. Athletes take part in sports activities for various motivations, such as need for companionship and rewards (Clancy et al., 2016). For collegiate student-atheletes, they participate in various sports competitions to obtain recognition and medals that could increase their confidence, enhance their popularity, and prepare for their career (Coakley, 2021). While enjoying the various benefits provided by universities (Condello et al., 2019), collegiate student-athletes have to fulfill the athletic role and academic role to deveop a sustainable career (Navarro et al., 2019; Stokowski et al., 2020; Fridley et al., 2023). These roles demand intensitve psysical training and practices, representing the university in sports competitions, while attending academic courses and keeping a minimum academic performance. Such requirements, together with concerns for injury, performance, fatigue, and finance, constitute the stressors that harm collegiate student-athletes’ well-being (Gill et al., 2017; Lopes Dos Santos et al., 2020; Wicker and Frick, 2020). For instance, many collegiate student-athletes often have poor academic backgrounds and learning habits, which, combined with heavy schedules and reduced study motivations, could harm their well-being (e.g., frustrations & low performance) (Rubin, 2016; Gill et al., 2017; Liang et al., 2021).

Moreover, collegiate student-athletes are challenged with adjustment to a new learning environment, managing new peer relationships, financial stress, and preparing for career development (Gross et al., 2018). Recently, the COVID-19 pandemic has added further medical and psychological pressure to collegiate student-athletes. In China, university on-campus teaching was cancelled and students took online learning from the start of 2020. University students had to take online learning, with limited outdoor activities and interpersonal communication; therefore, they are more likely to experience psychological stress (Zhan et al., 2021). Therefore, it is inferred that the stress and anxiety level of college students during the COVID-19 pandemic is generally high, especially for those who have not yet resumed school. Long-term negative emotions can easily lead to serious mental diseases such as cognitive impairment. First, athletic students having coronavirus often show no symptoms or mild symptoms, yet medical staff are not sure whether those students are safe to resume training and playing sports (Brito et al., 2021). Second, many athletic seasons are shortened and even canceled, with the training schedule uncertain, thus preventing them from maintaining the optimum physical conditions required for success in sports. Such worries can blend with the pressure to take enough courses and maintain good academic performance to safeguard their collegiate student-athlete identity at the university (Fogaca, 2021). These stressors could lead to developmental and psychological problems that harm collegiate student-athletes’ well-being. Therefore, understanding the factors that help collegiate student-athletes to improve well-being becomes important.

Previous studies have realized the importance of collegiate student-athletes’ cognitive, behavioral, and emotional regulation to cope with stressors (Leprince et al., 2018). While self-regulation could allow collegiate student-athletes to address stress (Dubuc-Charbonneau and Durand-Bush, 2015), other scholars remind that collegiate student-athletes need critical knowledge acquired through interactions with peers and coaches (Hague et al., 2021; Liang et al., 2021). University students’ knowledge sharing may involve sharing of internship information, discussing academic topics or concepts, and collaborating on academic assignments or team projects (Eid and Al-Jabri, 2016). For collegiate student athletes who spend most of time training, they need to frequently interact with peers (especially those non-athlete students) to learn how to solve academic questions and pass academic examinations. Lopez et al. (2020) empirically examined and confirmed that collegiate student-athletes’ personality traits could help reduce the impact of stressors (e.g., fears of uncertainty & losing scholarship due to abusive supervision from coaches) that harm their well-being. In particular, openness to experience as a personality trait is positively associated with stress regulation (Williams et al., 2009; Cooper et al., 2014). Openness to experience involves an individual’s sensitivity, curiosity, attention, and independent evaluation of to the environmental cues. Openness to experience allows individuals to recognize stressors, regulate physiological and emotional responses to stress, and initiate restorative mechanisms such as sleep (Williams et al., 2009) and social interactions.

Interactions with peers can happen through knowledge sharing, i.e., exchange of information, skills, and expertise among peers (including athletes and non-athletes) (Hosen et al., 2021). A student athlete’s effective learning requires knowledge sharing with athletic and non-athletic peers for their reciprocal favor of knowledge in an academic subject or a competition (Werner and Dickson, 2018). However, knowledge sharing is voluntary and can be impeded by factors such as lack of trust and good relationship (Jer Yuen and Shaheen Majid, 2007). This study follows the literature (Obrenovic et al., 2022) to examine how collegiate student-athletes’ personality helps overcome such difficulties; doing so could improve our knowledge on the individual factors can be nurtured. Moreover, coaches can influence collegiate student-athletes’ psychological states and health (Kavussanu et al., 2008). Feltz et al. (1999) examined the impact of coaching efficacy, i.e., the degree to which coaches have confidence about their abilities to influence the motivation, skills, and performance of athletes (Boardley, 2018). While coaching efficacy could suggest coaching behaviors such as athlete feedbacks and management tactics, scholars (Pulido et al., 2021) remind that athletes’ perceptions are critical to the evaluation of coaching effectiveness, which could shape collegiate student-athletes’ motivation and behaviors related to well-being. However, not much attention has been given to the moderating role of perceived coaching effectiveness in the context of knowledge sharing in universities.

Given the above gap, this study aims to answer: how does knowledge sharing with athletic and non-athletic peers influence collegiate student-athletes’ well-being? How does openness to experience influence an athlete student’s knowledge sharing behavior? How does perceived coaching effectiveness moderate the above relationship? In order to help understand the determinants of collegiate student-athletes’ wellbeing in the Chinese context, we draw on social learning theory to explain the role of collegiate student-athletes’ individual factors and perceived external supports from peers and coaches.

### Social learning theory

Collegiate student-athletes’ achievement of well-being through knowledge sharing and coaching can be explained by social learning theory. According to this study, individual behavior involves a continuous process under the influences of cognitive, behavioral, and environmental factors (Muro and Jeffrey, 2008). Moreover, social learning theory extends personality theories by explaining individuals’ behavioral changes when they join new environments and interact with new members (Ahammer, 1973). In particular, this theory integrates an individual’s personality, attention, and motivation to explain how personality traits affect individuals’ intention to interact with the new environment (Mischel, 1973), and how their observations and interaction with other actors in the environment influence their behaviors (Pinho et al., 2020).

For collegiate student-athletes who just begin university study, effective learning and stress-coping require them to interact with strangers (peers & coaches) in a new environment. The quality of interaction could be determined by collegiate student-athletes’ personality traits (e.g., openness to experience), which motivates them to share knowledge with peers to make up for knowledge gaps. Doing so could also enhance collegiate student-athletes’ social connectedness. However, some students may hold knowledge as their rare asset which is critical for competitive advantage (Ong et al., 2011). Previous studies (Jennex and Olfman, 2005; Jer Yuen and Shaheen Majid, 2007) have recognized the negative impacts of insufficient trust, weak relationship and weak motivation on students’ knowledge sharing activities. To address this puzzle, this study draws on social learning theory to examine how collegiate student-athletes’ openness to experience and their coaches’ encouragement collectively influence their knowledge sharing, which further affects their well-being.

### Knowledge sharing and collegiate student-athletes’ well-being

Knowledge could guide one’s logical thinking, modify behaviors, and improve communication (Lee and Choi, 2003), thereby enabling them to address difficult situations and improve well-being. The importance of knowledge sharing in learning contexts have been well recognized (Wu et al., 2011; Werner and Dickson, 2018). Knowledge sharing refers to the process where members reciprocally exchange knowledge to jointly specific problems (Jer Yuen and Shaheen Majid, 2007). Collegiate student-athletes may also need to acquire knowledge from professionals during internships and peers on campus. Such knowledge may include communication skills and collaboration habits, as well as experiences in addressing challenges, practical strategies to enhance athletic performances (Coffin et al., 2021; Piepiora et al., 2022). However, these precedents for collegiate student-athletes to solve problems in academic study and athletic career involve the active and voluntary sharing of knowledge with peers (Buchs et al., 2016; Yeşil and Hatunoğlu, 2019). Indeed, the sharing of critical information related to future career, procedures to apply for certain benefits or incidents, together with personally developed experience could allow collegiate student-athletes to creatively manage time and effectively address conflicts in training and study, thereby acquiring the sense of effectiveness in their own efforts. Moreover, social interactions with peers could help enhance mutual trust and relationship with peers, thereby giving students a sense of meaningfulness and creativity (Eid and Al-Jabri, 2016), which are critical for students to meet their needs for self-worth, thereby improving well-being.

Moreover, collegiate student-athletes often compete in various sports events with athletic peers who constitute a special social environment where they have to rely on each other as a group (e.g., university) to compete against other groups (e.g., universities) (Martin et al., 2014). In this case, collegiate student-athletes who compete as teams or individually against other universities may share information for their peers to perform better. Such proactive sharing of knowledge could earn social support and complementary knowledge from peers, relieve stress, and improve mental health, thereby improving well-being. As a result, we predict that collegiate student-athletes who are more willing to share athletic or academic knowledge with peers are likely to report reduced stress and improved well-being. Therefore, the following hypothesis can be developed.

*H1:* Knowledge sharing is positively associated with collegiate student-athletes’ well-being.

### Openness to experience and knowledge sharing

According to sports psychology researchers (Hoar et al., 2010; Leprince et al., 2018), the stressors that harm well-being come from the interactions between athletes and their environment. An important construct to examine individuals’ stress coping is personality, i.e., the patterns of thinking, feeling, and behaving that an individual consistently displays (Segerstrom and Smith, 2019). Personality traits (i.e., neuroticism, extraversion, openness to experience, agreeableness, and conscientiousness) can constantly demonstrate an individual’s behavioral patterns and thus has been widely adopted to interpret athlete performance (Piepiora, 2021a,b; Piepiora et al., 2021, 2022). Among the big-five personality traits, a widely proved significant predictor of subjective well-being is openness to experience (Dong and Ni, 2020; Ma et al., 2022; Tucaković and Nedeljković, 2022), i.e., an individual’s willingness to explore, tolerate, and consider new and unfamiliar ideas and experiences (McCrae and Costa, 1987).

Indeed, individuals with high degree of openness to experience may respond to new ideas curiously, with less stubbornness to old habits and more open to unfamiliar situations that enable them to avoid conflicts (Werner and Dickson, 2018). Collegiate student-athletes more open to experience are more likely to blend into various academic and athletic student groups and consider group members as partners; as such, they may regulate habits and attitudes when interacting with members (e.g., peers) of the new environment. Likewise, openness to experience may motivate collegiate student-athletes to develop and enhance reciprocal relationships with peers by sharing various types of knowledge (Werner and Dickson, 2018). That is, collegiate student-athletes more open to experience may share knowledge with peers in exchange for knowledge from peers to expand perspectives (Cui et al., 2022). Therefore, the following hypothesis can be predicted:

*H2:* Openness to experience is positively associated with collegiate student-athletes’ knowledge sharing.

Drawing on the above two hypotheses, we further suggest that knowledge sharing—as an important media for collegiate student-athletes to exchange ideas and knowledge with academic and athletic peers—mediates the positive relationship between openness to experience and collegiate student-athletes well-being. As mentioned above, achieving academic and athletic goals, and addressing problems in training are determinant for collegiate student-athletes’ well-being (Lopes Dos Santos et al., 2020). Collegiate students who are open to new experiences may find it easier to adjust to the new environment and develop positive perceptions of challenging situations (Schmiedeberg and Thönnissen, 2021). The adjustment process requires tolerance of differences and learning of complementary knowledge from peers. Collegiate student-athletes who are open to experience are motivated to share knowledge with peers in exchange for the required knowledge (e.g., avoiding injuries during training, & justifying for deadline extensions) that help address the stressors that affect their well-being. Thus, the following hypothesis is proposed:

*H3:* Knowledge sharing mediates the positive relationship between openness to experience and collegiate student-athletes’ well-being.

### Moderating effect of perceived coaching effectiveness

In addition to peers, coaches play critical roles in collegiate student-athletes’ learning outcomes such as motivation and performance (Amorose and Nolan-Sellers, 2016; Piepiora et al., 2022). Indeed, coaching involves a process where collegiate student-athletes develop abilities, confidence, and social connections through learning and behavioral change (Kavussanu et al., 2008; Sonesh et al., 2015). Coaches’ behaviors, instructing strategies and learning situations could all affect coaching results (Vinson et al., 2016). While the above studies recognize the impacts of coaching on collegiate student-athlete performance, other studies further suggest that those impacts often dependent on athletes’ perceptions and evaluations of coaches’ abilities and behaviors (Phillips and Jubenville, 2009; Vinson et al., 2016). Amorose and Nolan-Sellers (2016) have recognized and confirmed the moderating role of athletes’ perceptions of what coaches do and say on the relationship between coaching behavior and coaching effect. Drawing on the coaching efficacy model (Feltz et al., 1999), we further predict that collegiate student-athletes’ perception of coaching motivation, strategies, techniques and abilities could influence their subsequent behaviors. When collegiate student-athletes with openness to experience consider their coaches to be capable individuals who are determined to help them improve performance, they are more likely to follow coaches’ advice. Indeed, coaches are able to assess the various academic and athletic areas that may affect collegiate student-athletes’ career and serve as facilitator of mutual learning and knowledge sharing by modifying collegiate student-athletes’ attitude and behaviors (Provvidenza and Johnston, 2009). Therefore, coaches may facilitate knowledge sharing behavior of collegiate student-athletes with openness to experience, especially when they are perceived as effective. As such, we predict the following hypothesis:

*H4:* Perceived coaching effectiveness positively moderates the relationship between openness to experience and knowledge sharing.

**Figure 1:**
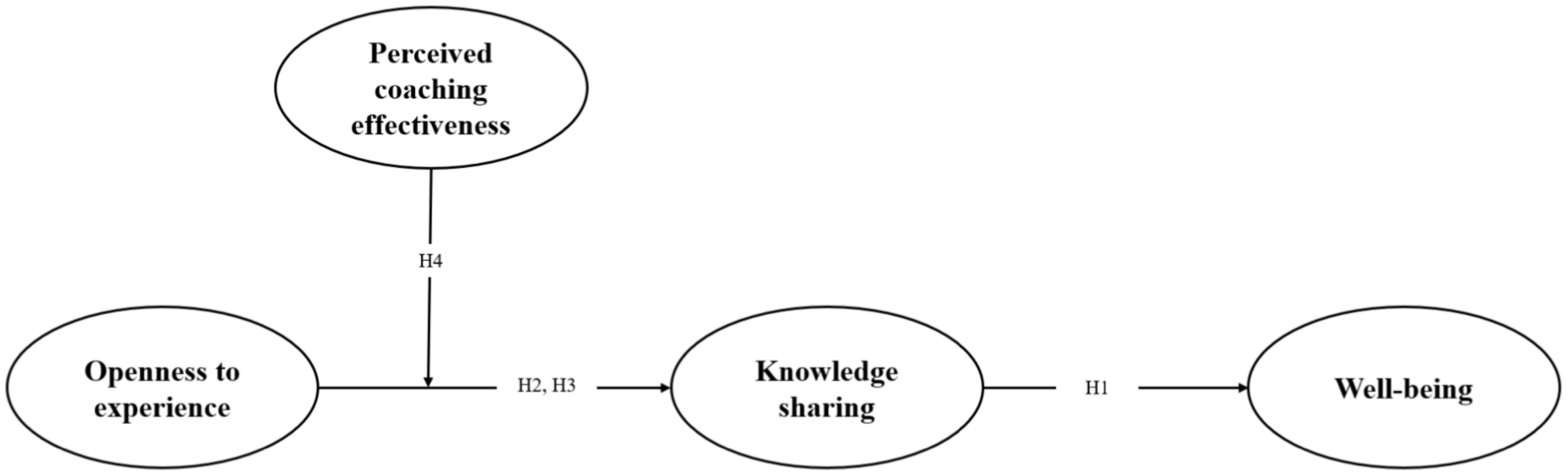
Conceptual Framework.

Figure 1 summarizes the above hypotheses.

## Methods

### Sampling

This study examined the hypothesized relationships by surveying collegiate student-athletes from Chinese universities. The survey was scrutinized and approved by the Academic Research Committee of Shenyang City University (Approval number: 12/2022). The survey as administered by the online survey platform ‘WENJUANXING’ (a questionnaire survey platform widely used in China. We received 512 responses; after excluding 28 invalid questionnaires, the sample for analysis consisted of 484 responses, or 94.53% of the total. 59.7% of the respondents were male (*n* = 289), freshman grade (year-one) collegiate student-athletes accounted for 43.8% (*n* = 212), and 81.4% of them (*n* = 394) were third-grade (year-three) student-athletes). The top three sports played by those collegiate student-athletes were basketball, badminton, and table tennis. Table 1 presents the demographic information of those respondents.

**Table 1:** Demographic description.

Demographic characteristics		Frequency	Percentage
Gender	Male	289	59.7%
	Female	195	40.3%
Age	Freshman grade	212	43.8%
	Sophomore grade	140	28.9%
	Junior grade	83	17.1%
	Senior grade	49	10.1%
Athlete status	First-grade athletes	3	0.6%
	Second-grade Athlete	87	18.0%
	Third-grade Athlete	394	81.4%
Sport	Basketball	95	19.6%
	Badminton	93	19.2%
	Table Tennis	81	16.7%
	Volleyball	74	15.3%
	Track and field	71	14.7%
	Football	60	12.4%
	Others	10	2.1%

### Measures

The questionnaire for this study included four constructs: athlete students’ well-being, openness to experience, knowledge sharing, and perceived coaching effectiveness. We translated each item into Chinese because our respondents are Chinese. To ensure semantic equivalence, we utilized the back-translation technique proposed by Chan and Pollard (2001). The items were first translated into Chinese by one author, and then translated back into English by a bilingual author. A 5-point Likert scale was used for each measurement item (see Appendix 1 for the measurement scales).

### Collegiate student-athletes’ well-being

Well-being was measured by a 9-item scale adapted from Dong and Ni (2020). The respondents were asked to evaluate their academic learning and campus life since they enrolled. The scale included nine items, such as ‘Boring—interesting’, ‘Useless—valuable’, ‘Lonely—animated’, ‘Empty-full’, and ‘Hopeless-hopeful’.

### Openness to experience

A 6-item scale to measure openness to experience was adapted from Dong and Ni (2020). Items include ‘I love to immerse myself in this new environment and to explore all things that are possible’, ‘I like to cultivate and develop new hobbies with different people’, and ‘I am fascinated by sports activities and academic learning’, ‘When I attend a new course or a new match, I sometimes feel very excited’, and ‘I am curious about a lot of things.’

### Knowledge sharing

Regarding the measurement of knowledge sharing, we adapted a 6-item scale from Eid and Al-jabri (2016) with modifications. Sample items are ‘I frequently observe other student-athletes to gain knowledge and information’, ‘I frequently offer advice to fellow student-athletes following matches’, and ‘I frequently share my experience or knowledge with other student-athletes’.

### Perceived coaching effectiveness

Collegiate student-athletes’ perceptions of coaching effectiveness were measured using an adapted version of the Coaching Efficacy Scale (CES; Feltz et al., 1999). This scale was adapted to measure athletes’ perceptions of coaching effectiveness in another study that considered the perceptions of athletes from various sports (Kavussanu et al., 2008). The CES consists of four subscales measuring motivation (7 items), game strategy (7 items), technique (6 items), and character-building effectiveness (4 items).

### Control variable

Based on the existing literature, we conducted analyses with several control variables, including gender, age, athlete status, and sport, which may influence collegiate student-athletes’ knowledge sharing and well-being (Eid and Al-Jabri, 2016).

### Data analysis

A measurement model was first established via confirmatory factor analysis (CFA), using AMOS 24.0, and then the hierarchical regression analysis was conducted by using SPSS 25.0. In order to avoid the problem of common method bias, we statistically tested the potential influence of common method bias using a Harman’s single-factor test to minimize potential common method bias (Podsakoff et al., 2003). We adopted PROCESS macro to test the mediating effect. The simple slopes of the moderating effect were plotted according to the suggestion by Aiken et al. (1991).

## Results

### Common method bias

Harman’s single-factor test was utilized to examine the problem of common method bias. Seven factors with eigenvalues greater than 1 were identified by the analysis, with the first factor explaining less than 40% of the variance (30.21% of 77.61%). Therefore, our findings provided no serious indications of common method variance (Podsakoff et al., 2003).

### Validity and reliability

The CFA was performed to assess the validity of each construct. The measurement model of this research included athlete students’ well-being, openness to experience, knowledge sharing, and perceived coaching effectiveness. In this research, perceived coaching effectiveness was a second-order construct (sub factors including motivation, game strategy, technique, and character building). The CFA results indicated that the data had a good fit to the measurement model. CMIN/DF = 2.276, root mean square error of approximation (RMSEA) = 0.051, root mean square residual (RMR) = 0.058, and comparative fit index (CFI) = 0.944 (Hair, 2009). Use of the CFA results to determine the reliability and validity of all items (Table 2) revealed that factor loading for all individual items of each construct in the model were over 0.7. The average variance extracted (AVE) of four constructs exceeded the 0.50 threshold suggested by Fornell and Larcker (1981). Composite reliability (CR) of each construct was over 0.8, which indicated that each construct had acceptable internal consistency. Overall, the four constructs within the proposed model had a satisfactory model fit. Thus, the convergent validity and reliability of the key constructs in this study was supported. Given these results, all four proposed constructs were applied in further analyses.

**Table 2:** Results of validity and reliability.

Construct	Item	STD. Estimate	CR	AVE	Cronbach’s Alpha
Perceived coaching effectiveness	Motivation	0.758	0.838	0.564	0.965
	Game Strategy	0.715			
	Technique	0.764			
	Character-building	0.766			
Openness to experience	OE1	0.837	0.947	0.747	0.946
	OE2	0.900			
	OE3	0.909			
	OE4	0.846			
	OE5	0.875			
	OE6	0.816			
Knowledge sharing	KS1	0.885	0.934	0.703	0.933
	KS2	0.850			
	KS3	0.853			
	KS4	0.855			
	KS5	0.797			
	KS6	0.787			
Well-Being	WB1	0.836	0.967	0.765	0.965
	WB2	0.867			
	WB3	0.872			
	WB4	0.877			
	WB5	0.837			
	WB6	0.834			
	WB7	0.925			
	WB8	0.938			
	WB9	0.880			

### Correlation and discrimination analysis

Table 3 presents the means, standard deviations, square roots of AVEs, and Pearson correlations for all of the key variables. There was a significant positive correlation between the four primary variables, which provided partial support for hypotheses. The square roots of AVEs were greater than their correlation coefficients with other factors that strongly support the discriminant validity (Fornell and Larcker, 1981).

**Table 3:** Results of correlation and discrimination analysis.

Variables	Mean	SD	Gender	Age	Athlete status	Sport	OE	KS	PCE	WB
Gender	1.40	0.49	–							
Age	1.94	1.01	−0.011	–						
Athlete status	2.81	0.41	0.005	0.055	–					
Sport	3.72	1.80	−0.015	−0.061	0.05	–				
OE	3.67	1.04	0.066	−0.046	0.008	−0.079	0.865			
KS	3.61	1.10	0.039	−0.093*	−0.043	−0.088	0.572^**^	0.839		
PCE	3.63	0.83	−0.015	0.019	0.013	−0.040	0.152^**^	0.173^**^	0.751	
WB	3.72	0.90	−0.049	−0.057	−0.020	−0.021	0.518^**^	0.413^**^	0.119^**^	0.875

*N* = 484, ^**^, *p* < 0.01; ^*^, *p* < 0.05; OE, openness to experience; KS, knowledge sharing; PCE, perceived coaching effectiveness; WB, well-being. The diagonal values are square roots of AVEs.

### Hypothesis testing

**Figure 2:**
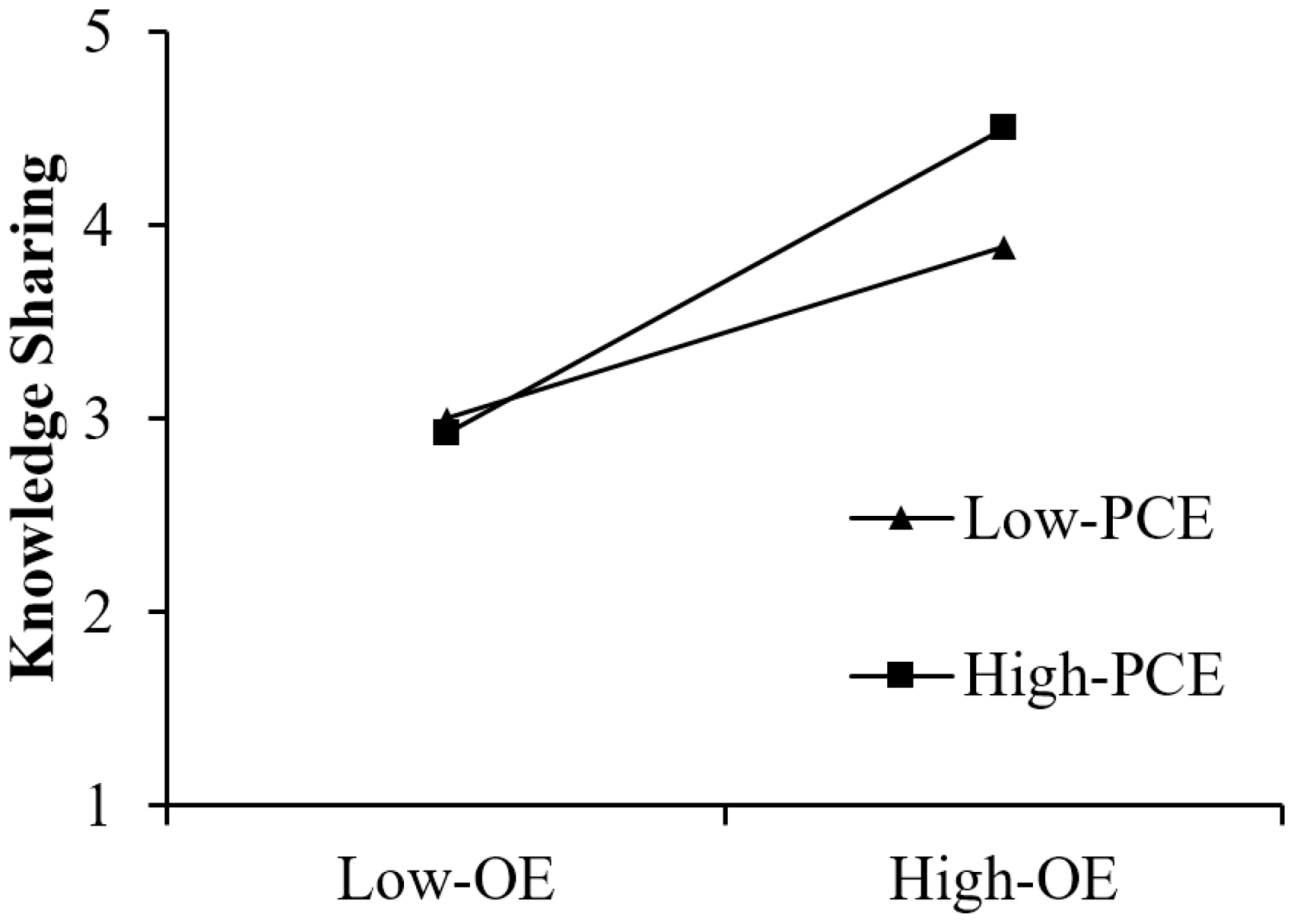
Moderating effect of PCE on OE & KS.

The direct and moderating effects are analyzed, and the results are presented in Table 4. To address multicollinearity, standardized values of the independent variables were used in all regression models (Toothaker et al., 1994). As can be seen, openness to experience (OE) had a positive impact on knowledge sharing (KS; β = 0.552, *p* < 0.05), the interaction effect between OE and perceived coaching effectiveness (PCE) had a positive impact on KS (β = 0.170, *p* < 0.05), and KS had a positive impact on WB (β = 0.415, *p* < 0.05). A simple slopes test presented in Figure 2 indicates that the moderating effect of PCE on the relationship between OE and KS is significant; that is, when PCE is at a high level (Mean + 1SD), the relationship between OE and KS is stronger than it is at a low level (Mean-1SD). Thus, H1, H2, and H4 were supported.

**Table 4:** Direct and moderating effects.

	KS			WB	
	Model 1	Model 2	Model 3	Model 4	Model 5
Gender	0.037	0.003	0.014	−0.050	−0.065
Age	−0.096^*^	−0.069	−0.065	−0.058	−0.018
Athlete status	−0.033	−0.042	−0.051	−0.015	−0.002
Sport	−0.092^*^	−0.043	−0.045	−0.025	0.013
OE		0.552^***^	0.556^***^		
PCE		0.089^*^	0.125^**^		
OE*PCE			0.170^***^		
KS					0.415^***^
R Square	0.020	0.343	0.371	0.007	0.175
Adjusted R Square	0.012	0.335	0.362	−0.002	0.167
F	2.428^*^	41.590^***^	40.092^***^	0.795	20.333^***^

*N* = 484, ^***^, *p* < 0.001; ^**^, *p* < 0.01; ^*^, *p* < 0.05; OE, openness to experience; KS, knowledge sharing; PCE, perceived coaching effectiveness; WB, well-being.

This study conducted bootstrap method to test the indirect mediating effect by using SPSS PROCESS template model 4. The indirect effect (see Table 5) of OE on WB via KS was 0.086 and bootstrapped 95% CI did not include zero (0.034, 0.137). Thus, H3 was supported.

**Table 5:** Result of the mediating effect.

Path	Effect	se	LLCI	ULCI
Direct effect (OE-WB)	0.362	0.041	0.282	0.441
Indirect effect (OE-KS-WB)	0.086	0.026	0.034	0.137

*N* = 484, OE, openness to experience; KS, knowledge sharing; WB, well-being.

## Discussion

Collegiate student-athletes are confronted with the dual requirements as students and athletes, which, combined with concerns for injury and fatigue, collectively form the stressors that harm their well-being (Lopes Dos Santos et al., 2020). Previous studies have recognized the importance of collegiate student-athletes’ cognitive, behavioral, and emotional regulations to cope with stressors (Dubuc-Charbonneau and Durand-Bush, 2015; Leprince et al., 2018; Coffin et al., 2021), yet neglected the source and impact of knowledge required to address those stressors, nor was the roles of personality trait (i.e., openness to experience) and perceived coaching effectiveness in stress coping evaluated in this context. As a result, this study draws on social learning theory to explain how collegiate student-athletes’ individual factors and perceived external supports from peers and coaches collectively help them meet various needs, thereby improving well-being.

Our research findings align with previous studies (Piepiora, 2021a,b; Piepiora et al., 2021, 2022) on the significance of personality in athletic careers. In particular, we confirmed the positive impact of openness to experience on collegiate student-athletes’ propensity to share knowledge; this indicates that openness to experience encompasses not only cognitive curiosity to explore new life experiences (Piepiora et al., 2022), but also a reduced resistance to changing established habits and an eagerness to acquire complementary knowledge from peers. Hence, we further substantiated the stress coping function of openness to experience among collegiate student-athletes. Furthermore, our results confirm that knowledge sharing is positively related to the well-being of collegiate student-athletes. Such a result concurs with the finding of (Piepiora et al., 2022) regarding the social nature of sport activities. We concur that knowledge sharing with peers enables collegiate student-athletes to acquire the social support and complementary knowledge that reshape their mindsets and behaviors to overcome difficulties during sport matches and career development.

Additionally, our results suggest that unlike professional athletes, collegiate student-athletes need to maintain not only athletic performance but also academic achievement. This requires collegiate student-athletes’ interactions non-athlete peers (Martin et al., 2014). Moreover, our results empirically proved the mediating role of knowledge sharing between openness to experience and well-being among collegiate student-athletes. In doing so, we answer the call from former researchers (Piepiora, 2021a) to investigate the impact of other actors in athletes’ social environment. Finally, we concur with former studies (Amorose and Nolan-Sellers, 2016; Piepiora et al., 2022) regarding the role of coaches in athletes’ performance and career development, as well as the importance of investigating stakeholders’ perceptions of coaching behaviors (Vinson et al., 2016). While these studies examine how coach-athlete relationship fosters athlete growth, this study highlights the importance of collegiate student-athletes’ perceptions of coaches’ motivations, strategies and abilities in their subsequent behaviors. Collegiate student-athletes who perceive their coaches to be capable are more likely to follow their instructions and suggestions to help other peers and share knowledge.

### Theoretical implications

This study examined the mechanism that allows collegiate student-athletes to address the various stressors that affect their well-being. In doing so, we shed lights on the student-athlete literature because former studies have primarily focused on university support and coaches as important sources for collegiate student-athletes to acquire knowledge (Coakley and Pike, 2009; Amorose and Nolan-Sellers, 2016; Egan, 2019). We particularly extended the athlete well-being literature (Dong and Ni, 2020) by unraveling the sources of knowledge that enables collegiate student-athletes to improve well-being. Our findings empirically supported the conceptual framework that guide more research efforts on the topic. Specifically, the framework illustrates how proactive knowledge sharing with peers, as an essential effort to stimulate peer reciprocation, could lead to improved collegiate student-athlete well-being; how personality (i.e., openness to experience) influences collegiate student-athletes’ knowledge sharing behavior. While we only focus on the collegiate student-athletes’ perspective, the conceptual framework also demonstrates that coaches (i.e., external factor) may positively influence collegiate student-athletes’ knowledge sharing behavior with peers in exchange for the complementary knowledge that improves well-being. Specifically, our results suggest the importance of perceived coaching effectiveness, which concurs with former scholars (Amorose and Nolan-Sellers, 2016) regarding the importance of coach suggestions on collegiate student-athlete behavior and performance. This suggests the need for further research unraveling the interactive impact of coaching and perception on collegiate student-athlete outcomes, especially those related to collegiate student-athlete well-being.

Finally, we found the role of openness to experience on knowledge sharing, reaffirming former scholars (Obrenovic et al., 2022), but went further to investigate its impact on collegiate student-athlete well-being. When universities develop and enforce requirements and rewards for collegiate student-athletes, they should recognize the importance of peer learning and encourage knowledge sharing among students. Moreover, candidates’ personalities should be considered when selecting and designing the various programs for collegiate student-athletes. For collegiate student-athletes with low degree of openness to experience, universities and coaches should consider additional sources of knowledge and support for them to cope with stressors and obtain well-being.

### Managerial implications

The study demonstrates the importance of knowledge sharing for collegiate student-athletes to address stressors that harm their well-being. To fulfill the dual requirements of academic study and athletic performance, collegiate student-athletes need to interact with academic and athletic peers for complementary knowledge that can help address challenges that affect well-being. Therefore, universities and collegiate student-athletes should realize the importance of peer learning (Eid and Al-Jabri, 2016), which serves as important avenues to well-being improvement. Moreover, peer learning is based on a reciprocal requirement, suggesting that collegiate student-athletes should proactively engage in knowledge sharing activities with peers to develop trust and good relationships which are essential to obtain the desired knowledge. However, this study finds that the knowledge-sharing behavior is influenced by collegiate student-athletes’ personality trait (i.e., openness to experience) which is stable and difficult to change (Cooper et al., 2014). In this case, we suggest that coaches and universities should stimulate a knowledge sharing atmosphere and consider rewards to students who are willing to share personally developed knowledge. Coaches should consider the long-term development of collegiate student-athletes, giving instructions not only on athletic training but also encouragement for academic learning. Meanwhile, we realize the importance of collegiate student-athlete’s perception in coaches’ suggestions. Coaches should consider clearly explaining their motivation, strategies, techniques, and competence to collegiate student-athletes; doing so is important, as collegiate student-athletes’ perception of coaching effectiveness could facilitate knowledge sharing behavior (Provvidenza and Johnston, 2009).

### Limitations and future research

Despite the above-mentioned implications, this study still has some limitations. First, we only examined the role of openness to experience in collegiate student-athlete well-being. Future studies could include more personality traits regarding their impacts on the hypothesized relationships, although investigating an exhaustive list of personality traits proves to be challenging. Second, this study aims to focus on a collegiate student-athlete perspective to investigate their perceptions of external impact (i.e., coaching effectiveness), as a collegiate student-athlete’s motivation for knowledge sharing hinges on his or her perceptions of external factors (i.e., coaching effectiveness & peer interaction). Future studies could include both internal and external factors to investigate antecedents of collegiate student-athlete well-being. Third, the antecedents of collegiate student-athlete well-being were examined using cross-sectional data. Future studies could consider mining texts from digital platforms to develop a richer and more refined understanding of the underlying mechanisms that affect collegiate student-athlete well-being.

## Conclusion

Drawing on social learning theory, this study adopts the collegiate student-athlete’s perspective to examine the hypothesized impacts of openness to experience, knowledge sharing, and perceived coaching effectiveness on collegiate student-athlete well-being. We empirically confirmed that knowledge sharing, an important antecedent of collegiate student-athlete well-being, is positively determined by collegiate student-athletes’ personality (i.e., openness to experience). We also confirmed the mediating role of knowledge sharing between openness to experience and well-being; that is, collegiate student-athletes with openness to experience may improve share knowledge with peers in exchange for complementary knowledge that enable them to improve well-being. Finally, perceived coaching effectiveness positively moderated the relationship between openness to experience and knowledge sharing. That is, perception of coaching effectiveness enhances collegiate student-athletes with openness to experience to follow coach suggestions to share knowledge with peers.

## Data availability statement

The raw data supporting the conclusions of this article will be made available by the authors, without undue reservation.

## Ethics statement

The studies involving human participants were reviewed and approved by Shenyang City University. The patients/participants provided their written informed consent to participate in this study.

## Author contributions

PN conceived the design of this research, planned the procedure, carried out data collection. LF drafted and revised the manuscript. All authors contributed to the article and approved the submitted version.

## Conflict of interest

The authors declare that the research was conducted in the absence of any commercial or financial relationships that could be construed as a potential conflict of interest.

## Publisher’s note

All claims expressed in this article are solely those of the authors and do not necessarily represent those of their affiliated organizations, or those of the publisher, the editors and the reviewers. Any product that may be evaluated in this article, or claim that may be made by its manufacturer, is not guaranteed or endorsed by the publisher.
