# Supplementary material for: Improving collegiate student-athletes’ well-being: exploring the roles of openness to experience, knowledge sharing and perceived coaching effectiveness
Source: Front Psychol. 2023 Jul 27;14:1191622. doi: 10.3389/fpsyg.2023.1191622 (PMC10413552; doi:10.3389/fpsyg.2023.1191622)
Supplement: Supplementary file 1 [file Data_Sheet_1.docx]

**Appendix 1: Measurement scales**

**Collegiate student-athletes’ wellbeing**

- S1: Boring—interesting
- S3: Useless—valuable
- S4: Lonely—animated
- S5: Empty—full
- S6: Hopeless—hopeful
- S7: Disappointing—rewarding
- S8: Life has not given me any chance—life is too good for me
- S9: Satisfactory—unsatisfactory

**Openness to experience**

- O1: I love to immerse myself in fantasies and to explore all things that is possible.
- O2: I like to cultivate and develop new hobbies.
- O3: I am fascinated by the beauty of sports.
- O4: When I participate in news about different sport activities, I sometimes feel very excited.
- O5: I am curious about a lot of sports.
- O6: I am stable and not easily upset by new experiences.

**Knowledge sharing**

- K1: I frequently observe other student-athletes to gain knowledge and information.
- K2: I frequently offer advice to fellow student-athletes following matches.
- K3: I frequently share my experience or knowledge with other student-athletes.
- K4: I spend some time with other student-athletes to exchange experiences.
- K5: I provide my knowledge and useful experience at the request of other student-athletes.
- K6: I post useful tips and experiences on my social media to share with other student-athletes.

**Perceived coaching efficacy (P):**

**Motivation**

- P1: Help collegiate student-athletes maintain confidence in themselves.
- P2: Help collegiate student-athletes to build self-esteem.
- P3: Motivate collegiate student-athletes.
- P4: Help collegiate student-athletes not to be overly confident when performing well.
- P5: Build the self-confidence of his collegiate student-athletes.
- P6: Build team confidence.
- P7: Motivate collegiate student-athletes to compete against a weak opponent.

**Game Strategy**

- P8: Recognize opposing team’s strengths during competition.
- P9: Understand competitive strategies.
- P10: Adapt to different game situations.
- P11: Recognize opponents’ weaknesses during competition.
- P12: Make critical decisions during competition.
- P13: Maximize his strengths during competition.
- P14: Adjust his game strategy to fit his talent.

**Technique**

- P15: Demonstrate the skills of his sport.
- P16: Coach individual athletes on technique.
- P17: Develop athletes’ abilities.
- P18: Recognize talent in athletes.
- P19: Detect skill errors.
- P20: Teach the skills of his sport.

**Character Building**

- P21: Instill an attitude of good moral character.
- P22: Instill an attitude of fair play among his athletes.
- P23: Promote good sportsmanship.
- P24: Instill an attitude of respect for others.
